# Decellularized Colorectal Cancer Matrices as Bioactive Scaffolds for Studying Tumor-Stroma Interactions

**DOI:** 10.3390/cancers14020359

**Published:** 2022-01-12

**Authors:** Ângela Marques-Magalhães, Tânia Cruz, Ângela Margarida Costa, Diogo Estêvão, Elisabete Rios, Pedro Amoroso Canão, Sérgia Velho, Fátima Carneiro, Maria José Oliveira, Ana Patrícia Cardoso

**Affiliations:** 1 i3S-Institute for Research and Innovation in Health, University of Porto, 4200-135 Porto, Portugal; angela.magalhaes@i3s.up.pt (Â.M.-M.); tcruz@i3s.up.pt (T.C.); angela.amorimcosta@ineb.up.pt (Â.M.C.); destevao@i3s.up.pt (D.E.); culex.rios@gmail.com (E.R.); svelho@i3s.up.pt (S.V.); fcarneiro@ipatimup.pt (F.C.); mariajo@ineb.up.pt (M.J.O.); 2 INEB-Institute of Biomedical Engineering, University of Porto, 4200-135 Porto, Portugal; 3 ICBAS-School of Medicine and Biomedical Sciences, University of Porto, 4050-313 Porto, Portugal; 4 IPATIMUP-Institute of Pathology and Molecular Immunology, University of Porto, 4200-135 Porto, Portugal; 5 Department of Pathology, Faculty of Medicine, University of Porto, 4200-319 Porto, Portugal; pedro.a.canao@gmail.com; 6 Department of Pathology, Centro Hospitalar Universitário São João, 4200-319 Porto, Portugal

**Keywords:** colorectal cancer, extracellular matrix, patient-derived scaffolds

## Abstract

**Simple Summary:**

Colorectal cancer is an increasingly prevalent disease that accounts for substantial mortality and morbidity and is responsible for an impaired quality of life. This scenario highlights the urgent need to better understand the biological mechanisms underlying colorectal cancer onset, progression and spread to improve diagnosis and establish tailored therapeutic strategies. Therefore, understanding tumor microenvironment dynamics could be crucial, since it is where the tumorigenic process begins and evolves under the heavy influence of the complex crosstalk between all elements: the cellular component (cancer cells and the non-malignant stromal cells), the non-cellular component (extracellular matrix) and the interstitial fluids. Bioengineered models that can accurately mimic the tumor microenvironment are the golden key to comprehending disease biology. Therefore, the focus of this review addresses the advanced 3D-based models of the decellularized extracellular matrix as high-throughput strategies in colorectal cancer research that potentially fill some of the gaps between in vitro two-dimensional and in vivo models.

**Abstract:**

More than a physical structure providing support to tissues, the extracellular matrix (ECM) is a complex and dynamic network of macromolecules that modulates the behavior of both cancer cells and associated stromal cells of the tumor microenvironment (TME). Over the last few years, several efforts have been made to develop new models that accurately mimic the interconnections within the TME and specifically the biomechanical and biomolecular complexity of the tumor ECM. Particularly in colorectal cancer, the ECM is highly remodeled and disorganized and constitutes a key component that affects cancer hallmarks, such as cell differentiation, proliferation, angiogenesis, invasion and metastasis. Therefore, several scaffolds produced from natural and/or synthetic polymers and ceramics have been used in 3D biomimetic strategies for colorectal cancer research. Nevertheless, decellularized ECM from colorectal tumors is a unique model that offers the maintenance of native ECM architecture and molecular composition. This review will focus on innovative and advanced 3D-based models of decellularized ECM as high-throughput strategies in colorectal cancer research that potentially fill some of the gaps between in vitro 2D and in vivo models. Our aim is to highlight the need for strategies that accurately mimic the TME for precision medicine and for studying the pathophysiology of the disease.

## 1. Introduction

Colorectal cancer (CRC) is an increasingly prevalent disease that accounts for substantial mortality and morbidity and is responsible for an impaired quality of life and high financial resource consumption [1]. Despite advances in the development of less invasive screening and diagnostic approaches, approximately 25% of CRC patients are still diagnosed with a distant metastatic disease [2]. Currently, available therapies have not only limited the curative impact but also developed resistance, leading to poor prognosis and increased mortality rates [3]. In particular, immunotherapy has a limited application in CRC, being only recommended to patients with high microsatellite instable (MSI) tumors, which correspond to less than 15% of all CRC cases [4]. This scenario highlights the urgent need to better understand the biological mechanisms underlying CRC onset, progression and spread to improve CRC diagnosis and establish tailored therapeutic strategies. For that, a detailed understanding of the tumor microenvironment is fundamental, since it is where the tumorigenic process begins and evolves under the heavy influence of the complex crosstalk between the cellular component (cancer cells and the non-malignant stromal cells), the non-cellular component (extracellular matrix—ECM) and the interstitial fluids [5].

Over the last few years, the ECM has become a hot topic of research since this complex network of macromolecules is much more than a physical and stable structure providing support to tissues. The ECM is an extremely dynamic component of the TME [6] that modulates the behavior of both tumor and cancer-associated stromal cells through its particular biochemical and biomechanical properties [7]. During tumor development, the ECM is significantly altered, both structurally and in terms of composition, usually enabling cellular transformation, angiogenesis, inflammation, invasion and metastasis [8,9]. These tumor ECM alterations translate into dysfunctional biomechanical tissue properties with increased stiffness activating several cellular pathways, such as YAP/TAZ [10], TXNIP [11], Rho/Rock-PTEN [12], PI3K-AKT [13], GSK3β [14] and AMPK [15,16].

Considering the relevant role of this cellular–acellular communication, several efforts have been made to develop new CRC models that accurately mimic the interconnections within the TME to understand the disease [17,18,19,20,21,22,23]. Until now, most cancer research has been performed with in vitro two-dimensional (2D) cell culture. However, it is known that cells behave differently in 2D and three-dimensional (3D) cultures, and that animal models do not truly represent the human tumor architecture [17]. Current 3D cancer models are now managing to bridge the gap between 2D monolayer cell lines, animal models and clinical research. There is an increasingly growing field for the development of 3D cell culture models that are able to closely recapitulate the TME landscape and screen anti-cancer drugs in CRC, such as bio-fabricated tissues [18], organotypic 3D-bioactive models [19] and cancer tissue-originated spheroids [20]. Among these, several reports have described interesting strategies using decellularized ECM from native tissues where the cellular component is removed and the tissue physiology is maintained [24,25,26].

Therefore, the focus of this review is to summarize the innovative and advanced 3D-based models of CRC, with a special highlight on the decellularization-based models, which offer the intrinsic native properties of the ECM to accurately resemble and reconstruct the TME to study CRC biology and drug discovery.

## 2. Colorectal Cancer

CRC is the most frequently diagnosed gastrointestinal neoplasia, affecting the colon and rectum [27]. It is ranked as the third most common incident and the second deadliest neoplasia worldwide. In 2020, there were 1,931,590 newly diagnosed CRC cases and 935,173 deaths, nearly 10% of all new cancer cases and deaths reported annually [28].

CRC is a highly complex and heterogeneous disease from both histopathologic and molecular standpoints [29]. Disease etiology involves genetic and environmental factors leading to hereditary syndromes or sporadic CRC. Pre-malignant tumors usually arise sporadically through well-known sequences of genetic and epigenetic alterations [30]. The genes affected and the order by which the alterations occur are directly linked to distinct pathways of carcinogenesis, leading to the development of tumors with different types of genetic instabilities and ultimately resulting in differential disease prognosis and therapy responses [31,32]. Most cancers arise from a pre-neoplastic lesion that can eventually evolve into malignant disease. In general, this precursor lesion can follow two major pathways: (i) the traditional adenoma–carcinoma pathway (70–90% of CRC cases) that begins upon APC mutation, followed by RAS activation or TP53 loss of function, or (ii) the serrated neoplasia pathway (10–20% of CRC cases), which is associated with RAS and RAF mutations and a CpG island methylation phenotype, leading to either microsatellite stable or unstable cancers [33]. Chronic inflammation is a widely recognized risk factor, and in fact, patients with inflammatory bowel diseases, such as ulcerative colitis and Crohn’s disease, have an increased risk of developing CRC [34].

CRC complexity and evolution are not solely dependent on an accumulation of genetic modifications in malignant cells. Currently, it is widely accepted that the microenvironment plays fundamental roles, not only in tumorigenesis, but also in controlling progression and dictating CRC prognosis [35]. In fact, tumorigenesis is a complex and dynamic process that develops within an intricate context of cellular (cancer cells, cancer-associated fibroblasts, immune cells, endothelial cells and other tumor-infiltrating cells) and acellular (ECM and ECM-associated molecules) components that interact and influence each other [36]. Tumor-associated macrophages (TAM) are the most abundant immune cells in solid tumors and are reported to play key roles in disease progression, namely in ECM remodeling, tumor metabolism, angiogenesis, invasion and metastasis [37]. In CRC, however, there is still controversy concerning the positive and negative effects of TAM. In some reports, high TAM infiltration is correlated with a worse outcome [38] and CRC progression [39], while others associate high macrophage infiltration with a lower liver metastasis [40] and an improved survival rate [41].

Due to the high heterogeneity of these types of tumors, a new classification system was recently proposed based on consensus molecular subtypes (CMS) that reflect significant biological differences: CMS1 (MSI Immune), CMS2 (Canonical), CMS3 (Metabolic) and CMS4 (Mesenchymal) [42]. Acknowledging the relevance of the TME, this classification includes features such as immune and stromal infiltration. Moreover, the primary tumor location also influences the predisposition for the formation of CRC from some CMS instead of others. While tumors from CMS1 and CMS3 are more likely to develop in the right-sided colon, tumors from CMS2 and CMS4 typically arise in the left-sided colon and rectum [33,36].

These features highlight the complexity of the disease and have to be considered in strategies aiming to model and study CRC.

## 3. The Role of the Extracellular Matrix in Colorectal Cancer Progression

**Figure 1:**
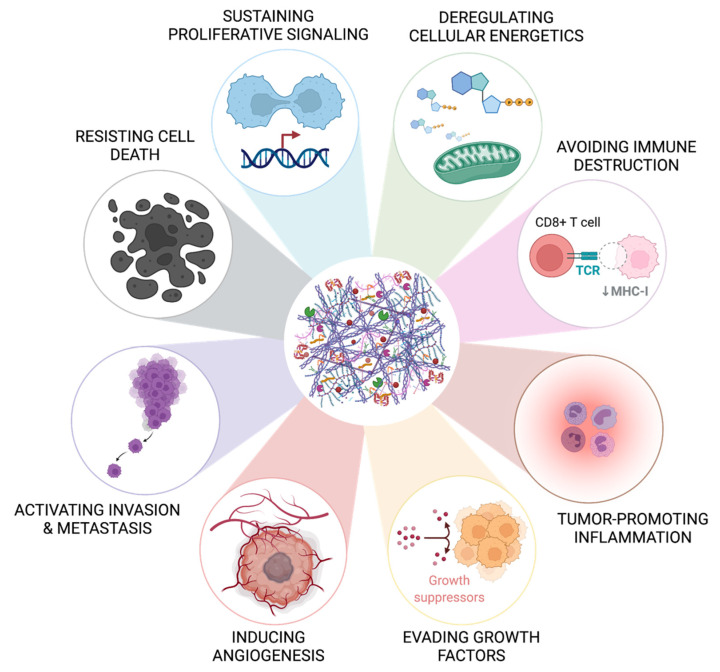
The extracellular matrix contributes to the hallmarks of cancer. The bioactive role of the extracellular matrix has been described to be involved in eight of the ten well-known hallmarks of cancer reported by Hanahan and Weinberg in 2011 [8], namely in resisting cell death, sustaining proliferative signaling, deregulating cellular energetics, avoiding immune destruction, tumor-promoting inflammation, evading factors, inducing angiogenesis, and activating invasion and metastasis. The influence of the extracellular matrix in the other two hallmarks (genome instability and mutation and enabling replicative immortality) is not yet fully characterized in CRC. Created with BioRender.com (accessed on 10 November 2021).

Cells composing the TME are within an elaborate and active network of ECM proteins, which provides a scaffold structure in which cells communicate and proliferate [9]. This ECM network is mainly composed of proteoglycans, glycoproteins, adhesive (i.e., fibronectin and laminin) and structural proteins (i.e., collagen and elastin), multiple metabolites, growth factors, and cytokines [9]. In tumor tissues, the ECM is considerably remodeled, losing structural organization and presenting an increased stiffness due to higher collagen content, crosslinking and fiber alignment [43]. Notably, the ECM is known to modulate distinct biological processes associated with cancer progression, namely increased cell proliferation, apoptosis and hypoxia resistance, invasion, metastasis, angiogenesis, cancer cell immune evasion, and stemness (Figure 1) [44,45,46,47,48]. This emphasizes the need to consider this acellular component when studying TME interactions and designing novel therapeutic strategies [19,26,49,50,51,52,53].

### 3.1. ECM Biochemical Features

In general, ECM proteins are common to both normal and malignant tissues. However, while these proteins are homogeneously distributed in normal tissues, they present an extremely irregular and heterogeneous distribution in tumors [50]. Among others, laminin, type I collagen, fibronectin and hyaluronic acid are both abundantly present in tumors but are also described as contributing to disease progression.

Type 1 collagen overexpression in tumor tissues has been implicated in the promotion of tumor growth, epithelial to mesenchymal transition (EMT), distant metastasis and increased stemness properties of CRC cells, through integrin α2β1 and the activation of PI3K/AKT/Snail and WNT/PCP signaling pathways [54,55,56]. Furthermore, it also reduces the E-cadherin/β-catenin axis activity and stimulates the expression of stem cell markers CD133 and BMI1 [56]. Additionally, higher collagen density inside the tumor compared with the surrounding stroma impairs immune cell migration into the tumor cell nest [57].

Laminin also displays an altered expression in tumors [19] and is involved in several cellular processes culminating in tumor progression and therapy resistance [45]. Laminin-α1 and α5 upregulation enrolls cancer-associated fibroblasts (CAFs), stimulates VEGFA production through the integrin α2β1-CXCR4 complex, and upregulates Notch signaling, promoting CRC growth, angiogenesis and metastatic spread [58,59]. Additionally, overexpression of laminin γ2 promotes CRC cell proliferation, migration and invasion [60], and laminin-α5 is associated with chemical resistance to chemotherapy with 5-fluorouracil (Figure 1) [61]. Interestingly, CRC disseminating metastasis frequently expresses high levels of laminin 521, which interacts with integrins α3β1 and α6β1 to promote cell invasion and self-renewal [62].

Hyaluronic acid, a non-sulfated glycosaminoglycan (GAG), is highly represented in tumors and constitutes an important component in promoting tumorigenesis, cell proliferation, migration and blocking apoptosis, namely by binding to CD44 and TLR4 [26,63].

Fibronectin is another vital component of the ECM that is upregulated in CRC and promotes cell proliferation through the NF-kB/p53 signaling pathway [64]. The extra domain A-containing fibronectin (EDA-FN) can increase VEGF expression associated with the PI3K/Akt-dependent pathway, promoting angiogenesis and metastasis [65]. CD133^+^/CD44^+^ colon cancer stem cells (CSCs) require EDA-FN binding to integrin α9β1 for sphere formation, and tumorigenic capacity by triggering the FAK/ERK/β-catenin signaling pathway [66].

A proteomic analysis of colorectal normal and tumor ECM revealed that collagen IV, V and XIV, fibrilin, emilin, vitronectin, laminin and endomucin have increased expression in tumor ECM, and that periostin, versican, thrombospondin-2 and tenascin were exclusively present in tumor tissue. Interestingly, when compared with available clinical gene expression array data, these signatures correlated with tumor progression and metastasis [51]. For example, the proteolysis of versican enhanced T cell infiltration independently of the mismatch repair status, while the fragments resulting from this proteolysis promoted the accumulation of dendritic cells, showing its chemotactic cues [67]. Tenascin-C induces αvβ3-mediated angiogenesis, promoting the development of colitis-associated cancer [68]. The levels of collagen XI (COL11A1) progressively increase along the tumor development through normal to adenoma to carcinoma cascade. High levels of COL11A1 are associated with an invasion signature and aggressive CRC phenotype through an upstream regulation performed by TGF-β. Therefore, increased COL11A1 expression is also associated with a poor survival rate and a poor prognosis in CRC patients [69]. Moreover, it was shown that tumor ECM induces an expression of genes associated with immune activation and regulation (EPX, PRG2 and DEFA3) and with cancer cell migration and motility (STOML2, HIF1a, and TNS4) [19].

The thrombospondin (THBS) family has been associated with the regulation of angiogenesis and cancer progression by controlling multiple physiological processes [70]. THBS-1 is highly expressed in the normal colon mucosa but is gradually lost in the adenoma–carcinoma cascade [71]. Furthermore, inhibition of THBS-1 promoted angiogenesis and tumor growth of colon carcinoma xenografts [72], and the expression of THBS-2 in CRC is correlated with inhibition of angiogenesis and lower hepatic metastasis [73], highlighting the importance of ECM regulation to impair tumor progression.

Each malignant tumor exhibits a specific proteoglycan molecular signature, which is closely associated with tumor differentiation and biological behavior [74]. In the case of CRC, proteoglycans play controversial roles in tumorigenesis. For example, Syndecan-1 and 2 exert tumorigenic effects in CRC by the activation of EGFR and MAPK pathways, culminating in disease dissemination and chemotherapy resistance [75,76]. However, it has also been reported that Syndecan-1 acts as a tumor suppressor by inhibiting cell growth and migration through the blockage of RAS/RAF/MEK/ERK and JAK1/STAT3 pathways in human CRC cells [77]. Besides, its depletion was associated with the activation of integrins and focal adhesion kinases (FAK)/Wnt signaling axis, which generate signals that potentiate tumor aggressiveness and stemness properties [78]. In turn, perlecan has been described to prompt tumor growth and angiogenesis in CRC [79].

Altogether, these studies highlight the influence of ECM composition in cellular modulation and disease progression. However, not only the biochemical composition of the ECM but also its biomechanical properties must be considered in terms of TME dynamics.

### 3.2. ECM Biomechanical Features

Besides alterations in composition, the ECM also suffers a structural rearrangement in the TME, with the alignment of fibers that ultimately contribute to an anisotropic configuration [80,81]. While an orderly ECM is crucial for regulation of cell behavior and tissue homeostasis, an anisotropic arrangement of the fibers is known to be a hallmark of malignancy [82].

Cell mechano-sensing translates biophysical forces into cellular responses, impacting several biological pathways, mechanisms and cell behavior. Solid tumors are constantly affected by mechanical stimuli, such as compression, matrix stiffness and fluid mechanics [83,84]. Tissue elasticity can be measured by shear wave elastography to discriminate between malignant and benign tissues, rheology and atomic force microscopy (AFM), which provide images of mechanical properties of biological samples with a high spatial resolution and result in elastographic data [85,86]. In the case of CRC, this is particularly interesting because the gastrointestinal tract is naturally submitted to more pronounced endogenous mechanical stress due to intestinal transit, leading to an extremely complex mechanical microenvironment [87]. As in other cancer types, CRC matrices are stiffer than the adjacent normal tissue [26,52], which is mainly due to collagen overexpression, deregulated crosslinking, and fiber rearrangement, along with increased GAG expression [43]. Anisotropic collagen is reported to be stiffer than isotropic collagen and occurs gradually from healthy to perilesional and CRC matrix [88]. Brauchle et al. demonstrated that this higher anisotropic collagen and altered fiber orientation in tumor tissues could rely on structural changes of proteins [43]. In CRC onset and progression, the pressure inside the tumor suddenly increases due to the combined compulsive proliferation of tumor cells with the inhibition of apoptosis [89]. This compressive stress is sufficient to cause flexion of the colonic crypt and subsequent deformation, budding and crypt fission [90,91]. Moreover, cancer-associated fibroblasts (CAFs) at the TME promote abnormal ECM deposition, exerting mechanical stress not only on the tumor itself, but also on adjacent normal tissues [92,93]. Recently, it was found that CRC matrix stiffening is not limited to the primary tumor location, but also to the uninvolved peripheric area (10–20 cm away from the neoplasm) [94]. In response to matrix stiffening, CAFs secrete activin A, a member of the TGF-β family, which strongly promotes a metastatic TME in the colon by inducing EMT and promoting cell migration and invasion, but also creates an immunosuppressive environment that favors cancer cell immune escape [95].

Lysyl oxidase (LOX) plays a vital role in this context by crosslinking collagen fibers, enhancing matrix compressing and stiffening in CRC, which then activates cell migration pathways [15]. Specifically, it can promote invasion and metastasis through β1-integrin activation and FAK/SRC signaling [96]. Increased levels of collagen crosslinking and linearized fibers may serve as migration highways on which tumor cells can travel [88]. Importantly, a dense ECM can also function as a reservoir of multiple growth factors and cytokines, creating a niche of signaling molecules [97] that enhance the motility of cancer cells and sustain malignant transformation [98,99]. LOX also initiates the Akt-VEGF pathway and stimulates the division of endothelial cells toward angiogenesis in CRC [100]. LOX-like protein 1 (LOXL1) negatively regulates the malignant progression of CRC by inhibiting Yes-associated protein (YAP) activity [101]. On the other hand, LOXL2 is a promoter of CRC invasion and metastasis [102], and its inhibition induces cell cycle arrest and apoptosis [103].

The protein cross-linking enzyme transglutaminase-2 (TG2) also has a role in modulating biomechanical properties through the formation of cross-links between glutamine and lysine sidechains of target proteins that are resistant to proteolytic degradation, exhibiting important pathophysiological functions [104]. In cancer, TG2 is involved in chemoresistance, apoptosis, invasion, migration, stemness and EMT [105]. Specifically in the CRC context, TG2 induces tissue stiffening mediated by fibroblasts, which are correlated with collagen fiber thickness and associated with a poor outcome in CRC patients [106]. An abnormal secretion of collagens and collagen-remodeling enzymes results in the re-organization of the ECM in the TME, and the fibrils become more linearized and compacted to thick collagen bundles, which are characteristic of tumor ECM [107].

Mechanical strains produced by external forces can initiate the expression of tumor-associated genes in CRC preneoplastic tissues [108]. Remarkably, a low strain of approximately 1.2 kPa, mimicking the applied stress on healthy tissues produced by early tumor growth, led to RET activation and downstream phosphorylation of β-catenin, increasing the expression of β-catenin-target genes and the formation of aberrant crypts. This behavior triggered abnormal cell growth that generated further mechanical stress. Interestingly, this study showed that the mechanical stimulation induced by cancerous tissues can directly modify the behavior of non-transformed adjacent cells toward a malignant behavior even in the absence of genetic mutations [92].

Matrix stiffness can also regulate the metastasis of CRC cells by SFK and MLCK through receptor-type tyrosine-protein phosphatase alpha (RPTPα) that senses mechanical stimulation [109]. HCT-8 cells, a grade I colon cancer cell line, progressively changed the morphology from an epithelial-like to a mesenchymal-like phenotype when cultured on gels with intermediate stiffness (21–47 kPa), accompanied by a decrease in E-cadherin in concert with cell–cell disassociation [110]. Later, the authors also showed that mesenchymal-like cells are remarkably more invasive than epithelial-like cells and exhibit high deformability, meaning that these aggressive cells are more likely to penetrate the epithelium of blood vessels. Additionally, these cells express molecular signatures linked to hypoxia and apoptosis resistance, and metastasis-associated genes [111]. Following detachment from the primary tumor, cancer cells intravasate into the blood vessels to disseminate. Nevertheless, few cells are able to resist the mechanical stress imposed by the blood flow [112]. To endure intravasation, circulation-associated shear stress and extravasation, CRC cells modify their mechanical properties by overexpressing integrin and integrin E-cadherin to increase the adhesion of tumor cells, as reviewed by Ciasca et al. [84]. Altogether, these findings suggest that the onset of metastasis may be linked to TME biomechanics and to the intracellular forces that allow complex cellular remodeling of the cytoskeleton.

Notably, contrary to normal ECM from the same patient, tumor ECM was able to polarize human macrophages into an M2-like phenotype, anti-inflammatory, and pro-tumor, with the expression of specific markers and the secretion of anti-inflammatory cytokines, such as CCL18 [26]. These macrophages were later able to promote CRC cell invasion through a CCL18-dependent mechanism [26].

Recently, the influence of matrix stiffness in treatment resistance has also gained attention. During chemotherapy, the dense and heterogenous structure of ECM in solid tumors are critical determinants for blood perfusion and interstitial transportation of the drug [113]. The expression of hyaluronic acid and sulfated GAGs was significantly elevated in CRC liver metastasis from patients treated with preoperative bevacizumab and chemotherapy. Additionally, anti-VEGF treatment was found to increase tumor stiffness [114].

Matrix stiffness also plays a role in radiation resistance. Ionizing radiation upregulates β1 integrins activating its downstream signals and increases the adhesion of CRC cells to collagen and fibronectin, contributing to the survival of cancer cells after treatment [115]. These experiments found mechanisms that can partially explain acquired resistance and provide ECM-related therapeutic targets for CRC treatment. Therefore, interest in developing therapeutic approaches targeting CRC matrix stiffness has been increasing [97,116].

Stiffening of the ECM progressively increases from early to later stages of CRC [117], emphasizing its role in the progression of the disease and its potential as a target for new anti-cancer therapies [81]. The mechanical landscape constitutes an important microenvironmental feature capable of regulating cancer cell proliferation, invasion and metastasis from a variety of cellular pathways. It is, therefore, imperative to include this parameter when studying tumor-stroma interactions.

## 4. Organotypic Models to Study ECM-CRC Cell Interactions

CRC 3D models have been recently reviewed, including hydrogels, patient-derived scaffolds, spheroids, organoids, microfluidic devices, and tumor and organ on-chip devices [22,24,25,118,119]. Three-dimensional organotypic models have shown a more realistic spatial conformation and polarization, increased cell junctions and cell-to-cell communication, as well as more accurate gene expression patterns reflecting truthfully in vivo pathophysiology, consequently increasing our understanding of CRC progression [118].

Biomaterial-based 3D organotypic models can be subdivided into scaffold-free or scaffold-based systems [120]. Scaffold-free systems allow cells to aggregate and proliferate without needing an adhesion platform, being cultured in, among others, hanging drop microplates, rotating devices, and static molds of agarose or collagen with low adhesion properties [120,121]. Recently, multicellular tumor spheroids have gained increased attention in cancer research due to their ability to aggregate and therefore mimic in vivo tumor characteristics of cellular communication, being particularly useful for studies involving radiotherapy, chemotherapy and resistance mechanisms [122,123]. The particular shape of multicellular tumor spheroids is achieved once the forces between cells are more significant than the forces between cells and the substrate on which they are platted, resulting in cellular aggregation. These are generally characterized by a central necrotic core with a decreased concentration of nutrients and oxygen, an inner layer of quiescent cells with a retained non-proliferative state and a front of proliferative cells [121]. Bauleth-Ramos et al. developed a successful CRC co-culture spheroid model of colon cancer cells, monocytes and human intestinal fibroblasts coated in an agarose mold to study the biocompatibility of nanoparticles and chemoimmunotherapy strategies for CRC treatment [121]. Nevertheless, these models also present some limitations, mainly their inability to reproduce the complexity of the human tissue architecture and matrix [120]. Therefore, scaffold-based 3D systems were developed to fill this gap by creating an artificial acellular matrix, in which cells are seeded and consequently proliferate. Natural biomaterials (i.e., collagen, fibrin, gelatin, agarose, alginate and chitosan) can be used to design scaffold-based 3D systems, having high biocompatibility and low toxicity when compared with synthetic biomaterials (i.e., polyethene, glycol and polycaprolactone) and ceramics (i.e., alumina, zirconia and bioglass) and that allow fine-tuning of the scaffold-based properties [120].

The most common 3D organotypic models for drug delivery and in situ tissue engineering are based on hydrogel-based scaffolds, frequently using natural polymers, such as collagen and alginate. These platforms are the most well-studied due to their low cost, low immunogenicity, versatility, biocompatibility and similarity to natural ECM [124]. These systems provide a biomimetic platform characterized by high water content (approximately 90%) and high permeability [125]. As an example, Luo and colleagues developed a hyaluronan-gelatin hydrogel for co-culture of CRC patient-derived organoids and patient-derived CAFs as a platform for drug screening [126]. However, one of the main drawbacks of hydrogels is their limited mechanical properties in the absence of covalent cross-linking [127]. To overcome this obstacle, efforts have been made to improve the mechanical and growth factor retention abilities of collagen hydrogels. A recent review of this subject described physical (e.g., firillogenesis and UV cross-linking), chemical (e.g., glutaraldehyde and genipin) and enzymatic (e.g., transglutaminase and LOX) cross-linking mechanisms to improve the mechanical properties of collagen hydrogels in terms of strength, durability, elasticity or compliance [124]. Growth factors are powerful molecules involved in various cellular processes and often function as signaling molecules between cells [128]. However, type I collagen does not have a high affinity and binding capacity for growth factors. Therefore, different strategies have been explored to improve the load capability of growth factors in scaffolds through direct loading, chemical cross-linking, electrostatic interactions and other carrier systems [124].

Three-dimensional bioprinting technology has gained increased relevance by allowing the standardization of the scaffold model between experiments [118]. This technique is based on the computer-assisted deposition of bioinks, which can include cells, hydrogels and decellularized matrices into specified 3D conformations [129]. Chen and colleagues developed a bionatural and slow-degrading collagen-polycaprolactone (PCL) bioprinted 3D CRC model, by co-culturing not only CAFs but also CRC cells and tumor-associated endothelial cells, mimicking the in vivo TME regarding proliferation, vascularization and adhesion [130]. The bioprinting scaffold-based 3D systems provide successful platforms for studying cell–cell interactions in CRC, but most of them still lack native components and fail to reproduce inter-patient ECM heterogenicity [118].

Even though hydrogel-based scaffolds and 3D bioprinting organotypic models are interesting tools to recreate the dynamics between major key players of CRC TME, they fail to incorporate fluidic dynamics between these components [131]. Scaffold-based 3D microfluidic systems composed of synthetic polymers, in which cells are cultured on chips coated with different ECM proteins, have been developed. These systems include different channels that allow a continuous diffusion of soluble factors and nutrients and the removal of waste products [131]. This approach allows the study of different cellular interactions in the same chip, considering the flow and hydrostatic pressure, mimicking more closely the native physiological conditions of the TME [131]. Very recently, Pinho and colleagues established a microfluidic device by culturing patient-derived CRC organoids on-a-chip. Through the continuous injection of culture medium, the authors allowed the proper growth and differentiation of the organoid, obtaining a good model for studying CRC modeling and drug screening applications [132]. However, this technology still involves extensive costs and optimization steps [131].

Altogether, the previously mentioned scaffold-based 3D systems represent suitable options for studying CRC TME interactions. Still, the exact native composition and structure of the ECM remains difficult to recreate reproducibly in vitro and, consequently, the recreation of the TME is highly restricted [19].

## 5. Decellularized Colorectal Cancer Matrices as Bioactive Scaffolds for Modeling the Tumor Microenvironment

Decellularized ECM from malignant tissues is gaining attention in the field of organotypic modeling of tumor-stroma interactions by successfully incorporating key biochemical and biophysical characteristics of the native TME [133,134,135]. Particularly, patient-derived scaffolds allow comparisons between the tumor and the normal adjacent tissues, as well as deliver the potential of a preclinical platform to test patient-specific responses to treatment therapies [136,137]. However, decellularized ECM as a biomimetic model for CRC research is just beginning to be explored (Table 1) [24,25].

Overall, decellularization protocols aim to eliminate all cell material while maintaining ECM architecture and biochemical components and are mainly a combination of physical, chemical and enzymatic methodologies [144]. The removal of cellular components without impairing the original ECM architecture, protein and glycoprotein distribution, and viscoelastic properties renders decellularization a reliable and attractive technique for studying ECM [19,26,49,52]. Several decellularization protocols and methodologies for evaluating the biochemical and biomechanical features of decellularized CRC ECM have been reported (Table 1). The lack of uniformity in these approaches obviously results in differences in decellularization efficiency, ECM characterization and ECM-mediated biological effects. Therefore, the uniformization of methodologies concerning CRC tissue decellularization would be essential for the reproducibility of results. Decellularized ECM can be cell-, animal-, or human- or patient-derived and have been applied as scaffolds for 3D cell cultures [145], bioprinting techniques for creating adaptable 3D structures, and as components in synthetic and natural cell culture platforms [146], providing bioactive ECM components in tunable constructs [147]. For example, the use of porcine jejunum decellularized matrix, derived from the patented Biological Vascularized Scaffold (DE:302014007893; BioVaSc^®^, proprietary knowhow Fraunhofer IGB) [148], is an interesting 3D in vitro colon model. This scaffold functions as an alternative biological colon-like structure with a mucosal tissue layer, including crypt, villi and a basement membrane, allowing the investigation of tumor cancer cell growth, EMT processes, cell invasion across the basement membrane, and metastization. Importantly, once this dynamic model is maintained in a bioreactor, the repopulation with CRC cells (i.e., SW480) and fibroblasts results in the formation of tumor-like clusters. Such data highlight the relevance of mimicking the tumor milieu to support malignant and stroma cell interactions [142]. Another exciting CRC biomimetic model was described by Alabi et al., who repurposed the use of mouse decellularized colon matrices, recellularized with HT-29 and HCT-116 human CRC cell lines, to investigate the crosstalk between ECM and cancer cell traits. Interestingly, mouse colon scaffolds were a better support for CRC cell HT-29 proliferation and differentiation than Matrigel^TM^. Moreover, HCT-116 cells displayed a higher rate of cell invasion when cultured in mouse colon cancer decellularized matrices than in cells cultured in wild-type mouse colon scaffolds or Matrigel^TM^ [143].

Three-dimensional ECM-hydrogels from decellularized human normal and tumor colon tissues have already been prepared through lyophilization, powdering and solubilization techniques and were useful for showing that tumor ECM components induced faster growth of HT-29 cells and their shift toward a glycolytic metabolism [52]. In an elegant study by Tian et al., organ-specific metastases were obtained by seeding CRC cells in a biomatrix coating composed of mouse lung and liver decellularized ECM [149]. In this system, 3D colonies were spontaneously formed and mimicked in vivo metastasis in terms of histological, molecular and phenotypic characteristics. Remarkably, these conditioned cancer cells exhibited tissue-specific tropism when injected into Nu/Nu mice.

Despite the undeniable utility and potential of these works that clearly show the impact of the matrix in CRC progression and metastasis, these approaches lack the native ECM architecture and mechanical properties that exhibit an active role in cell behavior [7,19].

**Figure 2:**
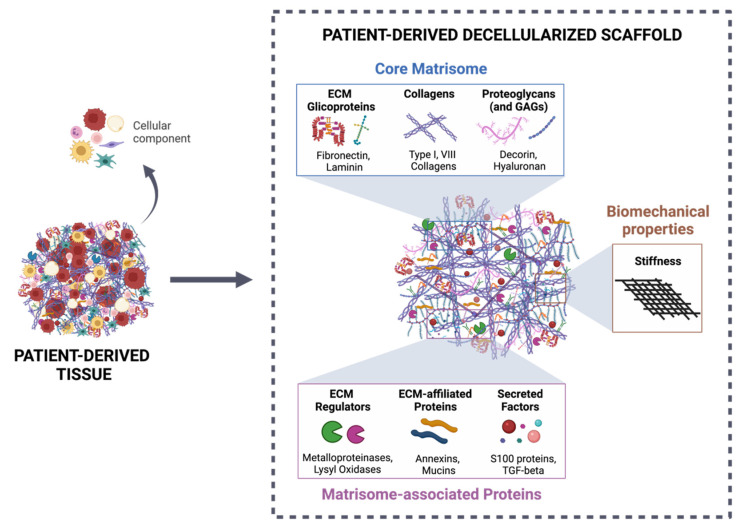
Patient-derived decellularized scaffold as a suitable tool for studying tumor–stroma interactions. The decellularization process of patient-derived tissue allows the efficient removal of the cellular component of the ECM, maintaining the 3D structure, as well as the biochemical categories (core matrisome and matrisome-associated proteins), according to Naba et al. [150], and biomechanical properties (stiffness). Created with BioRender.com (accessed on 10 November 2021).

In human CRC, strategies using decellularized ECM to study TME dynamics have been broadly focused on patient-derived scaffolds. Several protocols have been developed with the aim of efficiently removing cellular components from intestinal tissue while maintaining the architecture and biomechanical/biochemical features (Figure 2). These scaffolds have been successfully recellularized with different types of cells [19,26,49]. In 2016, Chen and Shuler described detailed procedures for establishing an organotypic human colon model from decellularized biopsy specimens, which were then recellularized with primary colon epithelial cells, endothelial cells, and fibroblasts [141]. This methodology allowed the identification of several genes that drive invasion in APC and KRAS mutated cells and demonstrated this model’s success in studying cancer biology under physiologically relevant conditions that include cell–matrix interactions and the spatial localization of multiple cell types, crucial in the TME context. 

In this field, approaches that consider paired CRC and normal adjacent tissue benefit from the direct comparison of samples from the same individual and allow the consideration of the role of tumor versus normal ECM on various cancer-associated activities and interactions with other TME components. Nevertheless, studies with access to this type of exceptionally valuable sample have been mainly restricted to recellularization with only one type of cell and require a further complex to create a structure that most trustworthily resembles the TME. Beyond proteomic and structural characterization of the decellularized ECM, reports showed that tumor ECM modulates IL-8 expression by HT29 cells [19] and that both HT-29 and HCT-116 cells reduced the sensitivity to 5-fluoracil when in a 3D ECM-setting [49,53]. In comparison with 2D cultures, HT-29 cells grown in CRC patient-derived scaffolds also displayed changes in the expression of genes and proteins related to proliferation and an increase in those concerning pluripotency and stemness [151].

From a different perspective, Pinto et al. [26] implemented a novel approach by studying the effect of human decellularized normal and tumor matrices derived from CRC patients’ surgical resections on the macrophage inflammatory signature. This work showed for the first time that, although derived from the same patient, normal and tumor matrices differently modulated the macrophage phenotype, with the last inducing an anti-inflammatory polarization, mimicking the immunosuppressive tumor microenvironment. Additionally, macrophages differentiated within tumor decellularized matrices stimulated CRC cell invasion through the expression of CCL18, an immunosuppressive chemokine identified as a key molecule in this process.

Decellularized tissues have also been applied in the study of the CRC metastatic process. D’Angelo and colleagues created a model with decellularized normal and primary tumor CRC tissue, as well as matched CRC liver metastasis with the aim of recapitulating this specific microenvironment in vitro [53]. This system demonstrated that HT-29 cells cultured in scaffolds derived from liver metastasis exhibit a higher EMT transition, a loss of E-cadherin and a higher vimentin expression, among other biological processes.

One of the major drawbacks of CRC patient-derived scaffolds is the limited amount of tumor tissue available from each individual, since it derives from biopsies or surgical resections, from which most tissue is required for further diagnostic molecular and histological characterization. Another question to keep in mind is that normal mucosa adjacent to the tumor, while often considered a healthy control from the same individual, in fact represents an intermediate state between normal and tumor tissues [152]. Despite collecting from at least 10 cm away from the tumor, a normal adjacent mucosa has a large number of differentially expressed genes in comparison with normal mucosa from healthy donors. This genetic disparity is essentially related to functions concerning the inhibition of matrix metalloproteinases (MMPs), cell adhesion molecules, TGF-β and integrin signaling pathways, inflammation, and cytokine–receptor interaction [153]. Interestingly, an integrative analysis of TCGA and GTEx RNA-seq data showed that normal adjacent tissue from the sigmoid colon is more similar to the tumor, while normal adjacent tissue from the transverse colon is more comparable to healthy tissue [152].

The recellularization of decellularized ECM also presents a few challenges, namely the choice of cell(s), the cells’ distribution within the scaffold and the reproducibility of recellularization efficiencies, even in samples from the same patient [154]. Concerning the specific case of CRC, strategies have been focused on small decellularized tissue fragments, static culture conditions, and recellularization with cancer cell lines. However, more sophisticated systems for mimicking the TME will require the inclusion of immune and stromal cells under dynamic cultures that will allow the flow of nutrients and molecules between the different compartments. To surpass the issue of spatial heterogeneity of the matrisome [155], tissue samples have to be representative of distinct tumor regions to avoid biasing experimental outcomes.

These are relevant topics to be considered when establishing an organotypic 3D model for CRC cancer with decellularized tissues, as well as for previously determining if there was previous neoadjuvant therapy. Still, the possibilities of these kinds of systems to incorporate ECM, cancer, stromal and immune cells will allow the study of the dynamic and complex crosstalks between the different components and recapitulate more closely the TME and, eventually, design strategies with potential for predicting clinical outcomes.

## 6. Conclusions

The future of cancer research relies on the implementation of translational 3D in vitro models that accurately mimic human tissues. Such models will foster an improved knowledge of cancer physiological and pathological processes, as well as facilitate drug discovery and screening. To scrutinize the molecular and cellular mechanisms in CRC, it is imperative that the approach to complex TME is recreated, namely the genetic, cell-to-cell, and cell–ECM cues that instruct cancer development and progression. To move forward on the study of TME interactions, decellularized colorectal matrices are attractive bioactive scaffolds, as they may be repopulated with different cell types and submitted to several soluble factors, pharmacological agents and/or radiation therapy.

We believe it is essential to standardize tissue-specific decellularization protocols according to tissue fragment size and to the intended final application. Additionally, a consensus on the methods to assess the decellularization efficiency, as well as the structural characterization of the decellularized ECM is also required. Until now, there is still limited information about effective long-term storage methodologies for these scaffolds, but some reports indicate that slow-freezing could provide an interesting solution [156,157]. We and others are currently gathering efforts to create a highly reproducible CRC TME organotypic model, including tumor ECM, cancer, immune and stromal cells. The possibilities for understanding transversal mechanisms of disease progression and/or creating pre-clinical platforms for drug testing and studying patient-specific resistance processes will be unlimited and provide powerful tools for novel therapeutic strategies.

In conclusion, it is widely accepted that accurate 3D cell culture models that consider interactions between CRC cells–TME–ECM are required for understanding disease biology and developing more advanced therapies regarding precision cancer medicine.

## Figures and Tables

**Table 1:** Methods used for the decellularization and evaluation of biochemical/biomechanical properties of decellularized ECM from colorectal tissues.

ECM Sources	Decellularization Method	Biochemical Evaluation	Biomechanical Evaluation	REF
Cell-derived matrix HT-29 SW480 CCD-841-Com	-CHEMICAL 0.5% Triton X-100 20 mM NH4OH Ionic and nonionic surfactants	n/a	n/a	[138,139,140]
Human-derived tissue	CHEMICAL 5 mM EDTA 10% DMSO 1% Triton X-100 10 mM sodium cholate hydrate 50 mM Tris-HCl Centrifugal rotation Ionic and nonionic surfactants Mechanical mixing	-Cellular proteins (cytokeratin, vimentin) and stromal components (collagen IV, fibrinogen, hyaluronic acid): Immunohistochemistry -Actin: Western Blot -DNA content: SYBR agarose gel	-Architecture: HE -3D structure: FITC staining of ECMs	[50]
	CHEMICAL/ENZYMATIC 4% sodium deoxycholate 2000 kU DNase-I	-DNA content: DNeasy Blood & Tissue kit -Stromal components (GAGs, Col IV): PAS and Immunohistochemistry -Cellular proteins (Ki67, vimentin, E-cadherin, DAPI): Immunofluorescence	-Architecture: HE and Laminin -3D structure: SEM -Permeability: In-house developed permeability device	[49]
		-DNA content: DNeasy Blood & Tissue kit and 1% SYBRsafe agarose gel -Stromal components (GAGs, Col IV): PAS, Masson’s Trichrome, Immunohistochemistry and Alcian blue	-Architecture: HE, Gieson and Silver stains -3D structure: SEM	[19]
	PHYSICAL/CHEMICAL Freezing 2% SDC 1% Triton X-100 Physical disruption Ionic and nonionic surfactants	-Nucleic acids: HE -Collagens: SHG	-Stiffness: AMR -Topography: SHG	[52]
	CHEMICAL/ENZYMATIC 0.1% SDS 50 U/mL DNase-I Ionic surfactant	-Nucleic acids: DAPI -DNA content: PureLink Genomic DNA Mini Kit -Histomorphological analysis: HE and Masson’s Trichrome -Major ECM proteins (Collagens I and IV, Laminin, Fibronectin and Hyaluronic acid): Immunofluorescence	-Stiffness: Rheology -3D structure: SEM	[26]
	CHEMICAL 1% SDS 1% Triton X-100	-DNA content: Nanodrop -Major ECM proteins (GAGs, Collagen I, Laminin and fibronectin): Immunostaining -Cellular proteins: F-actin (cytoskeleton), DAPI and HE (nuclei acid)	-Structure and architecture: SEM and TEM	[141]
SISmuc (small intestine submucosa + mucosa from decellularized porcine jejunum)	CHEMICAL 4% SDS 200 U/mL DNase I-	n/e	n/e	[142]
Mice-derived tissue	CHEMICAL/ENZYMATIC 4% sodium deoxycholate 2000 kU DNase-I	-DNA content: Roche’s DNA isolation Kit and Quant-It PicoGreen dsDNA Assay -Nucleic acids: DAPI and HE -Major ECM proteins (Collagens I and IV, Fibronectin and Laminin): Immunofluorescence and Masson’s Trichrome	-Tensile testing: RSA-G2 solids analyzer	[143]

AMR: Active microrheology; AFM: Atomic force microscopy; SHG: Second harmonic generation; n/a: not applicable; HE: Hematoxylin & Eosin; PAS: Periodic acid-Schiff; SEM: Scanning electron microscopy; SDS: sodium dodecyl sulfate; GAG: Glycosaminoglycan; n/e: not evaluated.

## References

[1] Dubé C. (2012). Tackling colorectal cancer as a public health issue: What can the gastroenterologist do?. Can. J. Gastroenterol..

[2] Vatandoust S., Price T.J., Karapetis C.S. (2015). Colorectal cancer: Metastases to a single organ. World J. Gastroenterol..

[3] Choi Y., Sateia H.F., Peairs K.S., Stewart R.W. (2017). Screening for colorectal cancer. Semin. Oncol..

[4] Ganesh K., Stadler Z.K., Cercek A., Mendelsohn R.B., Shia J., Segal N.H., Diaz L.A. (2019). Immunotherapy in colorectal cancer: Rationale, challenges and potential. Nat. Rev. Gastroenterol. Hepatol..

[5] Balkwill F.R., Capasso M., Hagemann T. (2012). The tumor microenvironment at a glance. J. Cell Sci..

[6] Hynes R.O. (2009). The extracellular matrix: Not just pretty fibrils. Science.

[7] Pickup M.W., Mouw J.K., Weaver V.M. (2014). The extracellular matrix modulates the hallmarks of cancer. EMBO Rep..

[8] Hanahan D., Weinberg R.A. (2011). Hallmarks of Cancer: The Next Generation. Cell.

[9] Winkler J., Abisoye-Ogunniyan A., Metcalf K.J., Werb Z. (2020). Concepts of extracellular matrix remodelling in tumour progression and metastasis. Nat. Commun..

[10] Scott K.E., Fraley S.I., Rangamani P. (2021). A spatial model of YAP/TAZ signaling reveals how stiffness, dimensionality, and shape contribute to emergent outcomes. Proc. Natl. Acad. Sci. USA.

[11] Sullivan W.J., Mullen P.J., Schmid E.W., Flores A., Momcilovic M., Sharpley M.S., Jelinek D., Whiteley A.E., Maxwell M.B., Wilde B.R. (2018). Extracellular Matrix Remodeling Regulates Glucose Metabolism through TXNIP Destabilization. Cell.

[12] Park J.S., Burckhardt C.J., Lazcano R., Solis L.M., Isogai T., Li L., Chen C.S., Gao B., Minna J.D., Bachoo R. (2020). Mechanical regulation of glycolysis via cytoskeleton architecture. Nature.

[13] Peyrou M., Clément S., Maier C., Bourgoin L., Branche E., Conzelmann S., Kaddai V., Foti M., Negro F. (2013). PTEN protein phosphatase activity regulates hepatitis C virus secretion through modulation of cholesterol metabolism. J. Hepatol..

[14] You Y., Zheng Q., Dong Y., Wang Y., Zhang L., Xue T., Xie X., Hu C., Wang Z., Chen R. (2015). Higher Matrix Stiffness Upregulates Osteopontin Expression in Hepatocellular Carcinoma Cells Mediated by Integrin β1/GSK3β/β-Catenin Signaling Pathway. PLoS ONE.

[15] Levental K.R., Yu H., Kass L., Lakins J.N., Egeblad M., Erler J.T., Fong S.F.T., Csiszar K., Giaccia A., Weninger W. (2009). Matrix Crosslinking Forces Tumor Progression by Enhancing Integrin Signaling. Cell.

[16] Bays J.L., Campbell H.K., Heidema C., Sebbagh M., DeMali K.A. (2017). Linking E-cadherin mechanotransduction to cell metabolism through force-mediated activation of AMPK. Nat. Cell Biol..

[17] Baker B.M., Chen C.S. (2012). Deconstructing the third dimension—How 3D culture microenvironments alter cellular cues. J. Cell Sci..

[18] Devarasetty M., Dominijanni A., Herberg S., Shelkey E., Skardal A., Soker S. (2020). Simulating the human colorectal cancer microenvironment in 3D tumor-stroma co-cultures in vitro and in vivo. Sci. Rep..

[19] Piccoli M., D’Angelo E., Crotti S., Sensi F., Urbani L., Maghin E., Burns A., De Coppi P., Fassan M., Rugge M. (2018). Decellularized colorectal cancer matrix as bioactive microenvironment for in vitro 3D cancer research. J. Cell. Physiol..

[20] Kondo J., Ekawa T., Endo H., Yamazaki K., Tanaka N., Kukita Y., Okuyama H., Okami J., Imamura F., Ohue M. (2019). High-throughput screening in colorectal cancer tissue-originated spheroids. Cancer Sci..

[21] Di Modugno F., Colosi C., Trono P., Antonacci G., Ruocco G., Nisticò P. (2019). 3D models in the new era of immune oncology: Focus on T cells, CAF and ECM. J. Exp. Clin. Cancer Res..

[22] Sensi F., D’Angelo E., D’Aronco S., Molinaro R., Agostini M. (2018). Preclinical three-dimensional colorectal cancer model: The next generation of in vitro drug efficacy evaluation. J. Cell. Physiol..

[23] Xu R., Zhou X., Wang S., Trinkle C. (2021). Tumor organoid models in precision medicine and investigating cancer-stromal interactions. Pharmacol. Ther..

[24] Hoshiba T. (2019). Decellularized Extracellular Matrix for Cancer Research. Materials.

[25] Ferreira L.P., Gaspar V.M., Mano J.F. (2020). Decellularized Extracellular Matrix for Bioengineering Physiomimetic 3D in Vitro Tumor Models. Trends Biotechnol..

[26] Pinto M.L., Rios E., Silva A.C., Neves S.C., Caires H.R., Pinto A.T., Durães C., Carvalho F.A., Cardoso A.P., Santos N.C. (2017). Decellularized human colorectal cancer matrices polarize macrophages towards an anti-inflammatory phenotype promoting cancer cell invasion via CCL18. Biomaterials.

[27] Arnold M., Abnet C.C., Neale R.E., Vignat J., Giovannucci E.L., McGlynn K.A., Bray F. (2020). Global Burden of 5 Major Types of Gastrointestinal Cancer. Gastroenterology.

[28] Sung H., Ferlay J., Siegel R.L., Laversanne M., Soerjomataram I., Jemal A., Bray F. (2021). Global Cancer Statistics 2020: GLOBOCAN Estimates of Incidence and Mortality Worldwide for 36 Cancers in 185 Countries. CA Cancer J. Clin..

[29] Linnekamp J.F., Wang X., Medema J.P., Vermeulen L. (2015). Colorectal Cancer Heterogeneity and Targeted Therapy: A Case for Molecular Disease Subtypes. Cancer Res..

[30] Loree J.M., Pereira A.A.L., Lam M., Willauer A.N., Raghav K., Dasari A., Morris V.K., Advani S., Menter D.G., Eng C. (2018). Classifying Colorectal Cancer by Tumor Location Rather than Sidedness Highlights a Continuum in Mutation Profiles and Consensus Molecular Subtypes. Clin. Cancer Res..

[31] Fearon E.R., Vogelstein B. (1990). A genetic model for colorectal tumorigenesis. Cell.

[32] Velho S., Moutinho C., Cirnes L., Albuquerque C., Hamelin R., Schmitt F., Carneiro F., Oliveira C., Seruca R. (2008). BRAF, KRAS and PIK3CA mutations in colorectal serrated polyps and cancer: Primary or secondary genetic events in colorectal carcinogenesis?. BMC Cancer.

[33] Dekker E., Tanis P.J., Vleugels J.L.A., Kasi P.M., Wallace M.B. (2019). Colorectal cancer. Lancet.

[34] Kim E.R., Chang D.K. (2014). Colorectal cancer in inflammatory bowel disease: The risk, pathogenesis, prevention and diagnosis. World J. Gastroenterol..

[35] Liu Y., Cheng L., Li C., Zhang C., Wang L., Zhang J. (2021). Identification of tumor microenvironment-related prognostic genes in colorectal cancer based on bioinformatic methods. Sci. Rep..

[36] Colangelo T., Polcaro G., Muccillo L., D’Agostino G., Rosato V., Ziccardi P., Lupo A., Mazzoccoli G., Sabatino L., Colantuoni V. (2017). Friend or foe? The tumour microenvironment dilemma in colorectal cancer. Biochim. Biophys. Acta Bioenerg..

[37] Zhong X., Chen B., Yang Z. (2018). The Role of Tumor-Associated Macrophages in Colorectal Carcinoma Progression. Cell. Physiol. Biochem..

[38] Kwak Y., Koh J., Kim D.-W., Kang S.-B., Kim W.H., Lee H.S. (2016). Immunoscore encompassing CD3+ and CD8+ T cell densities in distant metastasis is a robust prognostic marker for advanced colorectal cancer. Oncotarget.

[39] Kang J.-C., Chen J.-S., Lee C.-H., Chang J.-J., Shieh Y.-S. (2010). Intratumoral macrophage counts correlate with tumor progression in colorectal cancer. J. Surg. Oncol..

[40] Zhou Q., Peng R.-Q., Wu X.-J., Xia Q., Hou J.-H., Ding Y., Zhou Q.-M., Zhang X., Pang Z.-Z., Wan D.-S. (2010). The density of macrophages in the invasive front is inversely correlated to liver metastasis in colon cancer. J. Transl. Med..

[41] Forssell J., Öberg A., Henriksson M.L., Stenling R., Jung A., Palmqvist R. (2007). High Macrophage Infiltration along the Tumor Front Correlates with Improved Survival in Colon Cancer. Clin. Cancer Res..

[42] Guinney J., Dienstmann R., Wang X., De Reyniès A., Schlicker A., Soneson C., Marisa L., Roepman P., Nyamundanda G., Angelino P. (2015). The consensus molecular subtypes of colorectal cancer. Nat. Med..

[43] Brauchle E., Kasper J., Daum R., Schierbaum N., Falch C., Kirschniak A., Schäffer T.E., Schenke-Layland K. (2018). Biomechanical and biomolecular characterization of extracellular matrix structures in human colon carcinomas. Matrix Biol..

[44] Stamenkovic I. (2003). Extracellular matrix remodelling: The role of matrix metalloproteinases. J. Pathol..

[45] Brassart-Pasco S., Brézillon S., Brassart B., Ramont L., Oudart J.-B., Monboisse J.C. (2020). Tumor Microenvironment: Extracellular Matrix Alterations Influence Tumor Progression. Front. Oncol..

[46] Andreuzzi E., Capuano A., Poletto E., Pivetta E., Fejza A., Favero A., Doliana R., Cannizzaro R., Spessotto P., Mongiat M. (2020). Role of Extracellular Matrix in Gastrointestinal Cancer-Associated Angiogenesis. Int. J. Mol. Sci..

[47] Nallanthighal S., Heiserman J.P., Cheon D.-J. (2019). The Role of the Extracellular Matrix in Cancer Stemness. Front. Cell Dev. Biol..

[48] Gordon-Weeks A., Yuzhalin A.E. (2020). Cancer Extracellular Matrix Proteins Regulate Tumour Immunity. Cancers.

[49] Sensi F., D’Angelo E., Piccoli M., Pavan P., Mastrotto F., Caliceti P., Biccari A., Corallo D., Urbani L., Fassan M. (2020). Recellularized Colorectal Cancer Patient-Derived Scaffolds as In Vitro Pre-Clinical 3D Model for Drug Screening. Cancers.

[50] Genovese L., Zawada L., Tosoni A.L., Ferri A., Zerbi P., Allevi R., Nebuloni M., Alfano M. (2014). Cellular Localization, Invasion, and Turnover Are Differently Influenced by Healthy and Tumor-Derived Extracellular Matrix. Tissue Eng. Part A.

[51] Naba A., Clauser K.R., Whittaker C.A., Carr S.A., Tanabe K.K., Hynes R.O. (2014). Extracellular matrix signatures of human primary metastatic colon cancers and their metastases to liver. BMC Cancer.

[52] Romero-López M., Trinh A., Sobrino A., Hatch M.M., Keating M.T., Fimbres C., Lewis D.E., Gershon P.D., Botvinick E.L., Digman M. (2017). Recapitulating the human tumor microenvironment: Colon tumor-derived extracellular matrix promotes angiogenesis and tumor cell growth. Biomaterials.

[53] D’Angelo E., Natarajan D., Sensi F., Ajayi O., Fassan M., Mammano E., Pilati P., Pavan P., Bresolin S., Preziosi M. (2020). Patient-Derived Scaffolds of Colorectal Cancer Metastases as an Organotypic 3D Model of the Liver Metastatic Microenvironment. Cancers.

[54] Zhang Z., Wang Y., Zhang J., Zhong J., Yang R. (2018). COL1A1 promotes metastasis in colorectal cancer by regulating the WNT/PCP pathway. Mol. Med. Rep..

[55] Wu X., Cai J., Zuo Z., Li J. (2019). Collagen facilitates the colorectal cancer stemness and metastasis through an integrin/PI3K/AKT/Snail signaling pathway. Biomed. Pharmacother..

[56] Kirkland S. (2009). Type I collagen inhibits differentiation and promotes a stem cell-like phenotype in human colorectal carcinoma cells. Br. J. Cancer.

[57] Salmon H., Franciszkiewicz K., Damotte D., Dieu-Nosjean M.-C., Validire P., Trautmann A., Mami-Chouaib F., Donnadieu E. (2012). Matrix architecture defines the preferential localization and migration of T cells into the stroma of human lung tumors. J. Clin. Investig..

[58] Mammadova-Bach E., Rupp T., Spenlé C., Jivkov I., Shankaranarayanan P., Klein A., Pisarsky L., Méchine-Neuville A., Cremel G., Kedinger M. (2018). Laminin α1 orchestrates VEGFA functions in the ecosystem of colorectal carcinoma. Biol. Cell.

[59] Gordon-Weeks A., Lim S.Y., Yuzhalin A., Lucotti S., Vermeer J.A.F., Jones K., Chen J., Muschel R.J. (2019). Tumour-Derived Laminin α5 (LAMA5) Promotes Colorectal Liver Metastasis Growth, Branching Angiogenesis and Notch Pathway Inhibition. Cancers.

[60] Huang D., Du C., Ji D., Xi J., Gu J. (2017). Overexpression of LAMC2 predicts poor prognosis in colorectal cancer patients and promotes cancer cell proliferation, migration, and invasion. Tumor Biol..

[61] Fukazawa S., Shinto E., Tsuda H., Ueno H., Shikina A., Kajiwara Y., Yamamoto J., Hase K. (2015). Laminin 3 expression as a prognostic factor and a predictive marker of chemoresistance in colorectal cancer. Jpn. J. Clin. Oncol..

[62] Qin Y., Shembrey C., Smith J., Paquet-Fifield S., Behrenbruch C., Beyit L.M., Thomson B.N.J., Heriot A.G., Cao Y., Hollande F. (2020). Laminin 521 enhances self-renewal via STAT3 activation and promotes tumor progression in colorectal cancer. Cancer Lett..

[63] Makkar S., Riehl T.E., Chen B., Yan Y., Alvarado D.M., Ciorba M.A., Stenson W.F. (2019). Hyaluronic Acid Binding to TLR4 Promotes Proliferation and Blocks Apoptosis in Colon Cancer. Mol. Cancer Ther..

[64] Yi W., Xiao E., Ding R., Luo P., Yang Y. (2016). High expression of fibronectin is associated with poor prognosis, cell proliferation and malignancy via the NF-κB/p53-apoptosis signaling pathway in colorectal cancer. Oncol. Rep..

[65] Xiang L., Xie G., Ou J., Wei X., Pan F., Liang H. (2012). The Extra Domain A of Fibronectin Increases VEGF-C Expression in Colorectal Carcinoma Involving the PI3K/AKT Signaling Pathway. PLoS ONE.

[66] Ou J., Deng J., Wei X., Xie G., Zhou R., Yu L., Liang H. (2013). Fibronectin extra domain A (EDA) sustains CD133+/CD44+ sub-population of colorectal cancer cells. Stem Cell Res..

[67] Hope C., Emmerich P.B., Papadas A., Pagenkopf A., Matkowskyj K.A., Van De Hey D.R., Payne S.N., Clipson L., Callander N.S., Hematti P. (2017). Versican-Derived Matrikines Regulate Batf3–Dendritic Cell Differentiation and Promote T Cell Infiltration in Colorectal Cancer. J. Immunol..

[68] Kawamura T., Yamamoto M., Suzuki K., Suzuki Y., Kamishima M., Sakata M., Kurachi K., Setoh M., Konno H., Takeuchi H. (2019). Tenascin-C Produced by Intestinal Myofibroblasts Promotes Colitis-associated Cancer Development Through Angiogenesis. Inflamm. Bowel Dis..

[69] Nallanthighal S., Heiserman J.P., Cheon D.-J. (2021). Collagen Type XI Alpha 1 (COL11A1): A Novel Biomarker and a Key Player in Cancer. Cancers.

[70] Stenina-Adognravi O., Muppala S., Gajeton J. (2018). Thrombospondins and remodeling of the tumor microenvironment. Vessel. Plus.

[71] Baek K.-H., Bhang D., Zaslavsky A., Wang L.-C., Vachani A., Kim C.F., Albelda S.M., Evan G.I., Ryeom S. (2013). Thrombospondin-1 mediates oncogenic Ras–induced senescence in premalignant lung tumors. J. Clin. Investig..

[72] Sundaram P., Hultine S., Smith L.M., Dews M., Fox J.L., Biyashev D., Schelter J.M., Huang Q., Cleary M.A., Volpert O. (2011). p53-Responsive miR-194 Inhibits Thrombospondin-1 and Promotes Angiogenesis in Colon Cancers. Cancer Res..

[73] Tokunaga T., Nakamura M., Oshika Y., Abe Y., Ozeki Y., Fukushima Y., Hatanaka H., Sadahiro S., Kijima H., Tsuchida T. (1998). Thrombospondin 2 expression is correlated with inhibition of angiogenesis and metastasis of colon cancer. Br. J. Cancer.

[74] Nikitovic D., Berdiaki A., Spyridaki I., Krasanakis T., Tsatsakis A., Tzanakakis G.N. (2018). Proteoglycans—Biomarkers and Targets in Cancer Therapy. Front. Endocrinol..

[75] Wang X., Zuo D., Chen Y., Li W., Liu R., He Y., Ren L., Zhou L., Deng T., Ying X.G. (2014). Shed Syndecan-1 is involved in chemotherapy resistance via the EGFR pathway in colorectal cancer. Br. J. Cancer.

[76] Hua R., Yu J., Yan X., Ni Q., Zhi X., Li X., Jiang B., Zhu J. (2020). Syndecan-2 in colorectal cancer plays oncogenic role via epithelial-mesenchymal transition and MAPK pathway. Biomed. Pharmacother..

[77] Wang S., Zhang X., Wang G., Cao B., Yang H., Jin L., Cui M., Mao Y. (2019). Syndecan-1 suppresses cell growth and migration via blocking JAK1/STAT3 and Ras/Raf/MEK/ERK pathways in human colorectal carcinoma cells. BMC Cancer.

[78] Kumar Katakam S.K., Tria V., Sim W.C., Yip G.W., Molgora S., Karnavas T., Elghonaimy E.A., Pelucchi P., Piscitelli E., Ibrahim S.A. (2021). The heparan sulfate proteoglycan syndecan-1 regulates colon cancer stem cell function via a focal adhesion kinase—Wnt signaling axis. FEBS J..

[79] Sharma B., Handler M., Eichstetter I., Whitelock J.M., Nugent M.A., Iozzo R.V. (1998). Antisense targeting of perlecan blocks tumor growth and angiogenesis in vivo. J. Clin. Investig..

[80] Crotti S., Piccoli M., Rizzolio F., Giordano A., Nitti D., Agostini M. (2017). Extracellular Matrix and Colorectal Cancer: How Surrounding Microenvironment Affects Cancer Cell Behavior?. J. Cell. Physiol..

[81] Emon B., Bauer J., Jain Y., Jung B., Saif T. (2018). Biophysics of Tumor Microenvironment and Cancer Metastasis—A Mini Review. Comput. Struct. Biotechnol. J..

[82] Provenzano P.P., Eliceiri K.W., Campbell J.M., Inman D.R., White J.G., Keely P.J. (2006). Collagen reorganization at the tumor-stromal interface facilitates local invasion. BMC Med..

[83] Avvisato C.L., Yang X., Shah S., Hoxter B., Li W., Gaynor R., Pestell R., Tozeren A., Byers S.W. (2007). Mechanical force modulates global gene expression and β-catenin signaling in colon cancer cells. J. Cell Sci..

[84] Ciasca G., Papi M., Minelli E., Palmieri V., De Spirito M. (2016). Changes in cellular mechanical properties during onset or progression of colorectal cancer. World J. Gastroenterol..

[85] Loft M.K., Pedersen M.R.V., Rahr H.B., Rafaelsen S.R. (2021). Can Ultrasound Elastography Discriminate between Rectal Adenoma and Cancer? A Systematic Review. Cancers.

[86] Zemła J., Danilkiewicz J., Orzechowska B., Pabijan J., Seweryn S., Lekka M. (2018). Atomic force microscopy as a tool for assessing the cellular elasticity and adhesiveness to identify cancer cells and tissues. Semin. Cell Dev. Biol..

[87] Basson M.D. (2003). Paradigms for Mechanical Signal Transduction in the Intestinal Epithelium. Category: Molecular, cell, and de-velopmental biology. Digestion.

[88] Nebuloni M., Albarello L., Andolfo A., Magagnotti C., Genovese L., Locatelli I., Tonon G., Longhi E., Zerbi P., Allevi R. (2016). Insight On Colorectal Carcinoma Infiltration by Studying Perilesional Extracellular Matrix. Sci. Rep..

[89] Li X.-L., Zhou J., Chen Z.-R., Chng W.-J. (2015). p53mutations in colorectal cancer- molecular pathogenesis and pharmacological reactivation. World J. Gastroenterol..

[90] Edwards C.M., Chapman S.J. (2007). Biomechanical Modelling of Colorectal Crypt Budding and Fission. Bull. Math. Biol..

[91] Nelson M.R., Howard D., Jensen O.E., King J.R., Rose F.R.A.J., Waters S.L. (2010). Growth-induced buckling of an epithelial layer. Biomech. Model. Mechanobiol..

[92] Fernández-Sanchez M.E., Barbier S., Whitehead J., Béalle G., Michel A., Latorre-Ossa H., Rey C., Fouassier L., Claperon A., Brullé L. (2015). Mechanical induction of the tumorigenic β-catenin pathway by tumour growth pressure. Nature.

[93] Kalluri R., Zeisberg M. (2006). Fibroblasts in cancer. Nat. Cancer.

[94] Despotović S.Z., Milićević N.M., Milošević D.P., Despotović N., Erceg P., Svorcan P., Schumacher U., Ullrich S., Mihajlović G., Kalem D. (2017). Remodeling of extracellular matrix of the lamina propria in the uninvolved human rectal mucosa 10 and 20 cm away from the malignant tumor. Tumor Biol..

[95] Bauer J., Emon M.A.B., Staudacher J.J., Thomas A.L., Zessner-Spitzenberg J., Mancinelli G., Krett N., Saif M.T., Jung B. (2020). Increased stiffness of the tumor microenvironment in colon cancer stimulates cancer associated fibroblast-mediated prometastatic activin A signaling. Sci. Rep..

[96] Baker A.M., Bird D., Lang G., Cox T.R., Erler J.T. (2013). Lysyl oxidase enzymatic function increases stiffness to drive colorectal cancer progression through FAK. Oncogene.

[97] Huang J., Zhang L., Wan D., Zhou L., Zheng S., Lin S., Qiao Y. (2021). Extracellular matrix and its therapeutic potential for cancer treatment. Signal Transduct. Target. Ther..

[98] Maeda S., Dean D.D., Gomez R., Schwartz Z., Boyan B.D. (2002). The First Stage of Transforming Growth Factor β1 Activation is Release of the Large Latent Complex from the Extracellular Matrix of Growth Plate Chondrocytes by Matrix Vesicle Stromelysin-1 (MMP-3). Calcif. Tissue Int..

[99] Zucker S., Vacirca J. (2004). Role of matrix metalloproteinases (MMPs) in colorectal cancer. Cancer Metastasis Rev..

[100] Baker A.-M., Bird D., Welti J.C., Gourlaouen M., Lang G., Murray G.I., Reynolds A.R., Cox T.R., Erler J.T. (2013). Lysyl Oxidase Plays a Critical Role in Endothelial Cell Stimulation to Drive Tumor Angiogenesis. Cancer Res..

[101] Hu L., Wang J., Wang Y., Wu L., Wu C., Mao B., Maruthi Prasad E., Wang Y., Chin Y.E. (2020). LOXL1 modulates the malignant progression of colorectal cancer by inhibiting the transcriptional activity of YAP. Cell Commun. Signal..

[102] Park P.-G., Jo S.J., Kim M.J., Kim H.J., Lee J.H., Park C.K., Kim H., Lee K.Y., Kim H., Park J.H. (2017). Role of LOXL2 in the epithelial-mesenchymal transition and colorectal cancer metastasis. Oncotarget.

[103] Cui X., Wang G., Shen W., Huang Z., He H., Cui L. (2018). Lysyl oxidase-like 2 is highly expressed in colorectal cancer cells and promotes the development of colorectal cancer. Oncol. Rep..

[104] Karsdal M.A., Nielsen M.J., Sand J.M., Henriksen K., Genovese F., Bay-Jensen A.-C., Smith V., Adamkewicz J.I., Christiansen C., Leeming D.J. (2013). Extracellular Matrix Remodeling: The Common Denominator in Connective Tissue DiseasesPossibilities for Evaluation and Current Understanding of the Matrix as More Than a Passive Architecture, but a Key Player in Tissue Failure. ASSAY Drug Dev. Technol..

[105] Huang L., Xu A.-M., Liu W. (2015). Transglutaminase 2 in cancer. Am. J. Cancer Res..

[106] Delaine-Smith R., Wright N., Hanley C., Hanwell R., Bhome R., Bullock M., Drifka C., Eliceiri K., Thomas G., Knight M. (2019). Transglutaminase-2 Mediates the Biomechanical Properties of the Colorectal Cancer Tissue Microenvironment that Contribute to Disease Progression. Cancers.

[107] Lu P., Weaver V.M., Werb Z. (2012). The extracellular matrix: A dynamic niche in cancer progression. J. Cell Biol..

[108] Whitehead J., Vignjevic D., Fütterer C., Beaurepaire E., Robine S., Farge E. (2008). Mechanical factors activate ß-catenin-dependent oncogene expression in APC1638N/+ mouse colon. HFSP J..

[109] Krndija D., Schmid H., Eismann J.-L., Lother U., Adler G., Oswald F., Seufferlein T., von Wichert G. (2010). Substrate stiffness and the receptor-type tyrosine-protein phosphatase alpha regulate spreading of colon cancer cells through cytoskeletal contractility. Oncogene.

[110] Tang X., Kuhlenschmidt T.B., Zhou J., Bell P., Wang F., Kuhlenschmidt M.S., Saif T.A. (2010). Mechanical Force Affects Expression of an In Vitro Metastasis-Like Phenotype in HCT-8 Cells. Biophys. J..

[111] Tang X., Kuhlenschmidt T.B., Li Q., Ali S., Lezmi S., Chen H., Pires-Alves M., Laegreid W.W., Saif T.A., Kuhlenschmidt M.S. (2014). A mechanically-induced colon cancer cell population shows increased metastatic potential. Mol. Cancer.

[112] Mehlen P., Puisieux A. (2006). Metastasis: A question of life or death. Nat. Cancer.

[113] Buyse M., Sargent D.J., Grothey A., Matheson A., De Gramont A. (2010). Biomarkers and surrogate end points—The challenge of statistical validation. Nat. Rev. Clin. Oncol..

[114] Rahbari N.N., Kedrin D., Incio J., Liu H., Ho W.W., Nia H.T., Edrich C.M., Jung K., Daubriac J., Chen I. (2016). Anti-VEGF therapy induces ECM remodeling and mechanical barriers to therapy in colorectal cancer liver metastases. Sci. Transl. Med..

[115] Meineke V., Gilbertz K.-P., Schilperoort K., Cordes N., Sendler A., Moede T., van Beuningen D. (2002). Ionizing Radiation Modulates Cell Surface Integrin Expression and Adhesion of COLO-320 Cells to Collagen and Fibronectin in Vitro. Strahlenther. Onkol..

[116] Liu C., Pei H., Tan F. (2020). Matrix Stiffness and Colorectal Cancer. OncoTargets Ther..

[117] Wei B., Zhou X., Liang C., Zheng X., Lei P., Fang J., Han X., Wang L., Qi C., Wei H. (2017). Human colorectal cancer progression correlates with LOX-induced ECM stiffening. Int. J. Biol. Sci..

[118] Castro F., Leite Pereira C.L., Helena Macedo M.H., Almeida A., José Silveira M.J., Dias S., Patrícia Cardoso A.P., José Oliveira M., Sarmento B. (2021). Advances on colorectal cancer 3D models: The needed translational technology for nanomedicine screening. Adv. Drug Deliv. Rev..

[119] Reidy E., Leonard N.A., Treacy O., Ryan A.E. (2021). A 3D View of Colorectal Cancer Models in Predicting Therapeutic Responses and Resistance. Cancers.

[120] Langhans S.A. (2018). Three-Dimensional in Vitro Cell Culture Models in Drug Discovery and Drug Repositioning. Front. Pharmacol..

[121] Bauleth-Ramos T., Feijão T., Gonçalves A., Shahbazi M.-A., Liu Z., Barrias C., Oliveira M.J., Granja P., Santos H.A., Sarmento B. (2020). Colorectal cancer triple co-culture spheroid model to assess the biocompatibility and anticancer properties of polymeric nanoparticles. J. Control. Release.

[122] Weiswald L.-B., Bellet D., Dangles-Marie V. (2015). Spherical Cancer Models in Tumor Biology. Neoplasia.

[123] Fitzgerald K.A., Malhotra M., Curtin C., Brien F.J.O., Driscoll C.M.O. (2015). Life in 3D is never flat: 3D models to optimise drug delivery. J. Control. Release.

[124] Sarrigiannidis S.O., Rey J.M., Dobre O., González-García C., Dalby M.J., Salmeron-Sanchez M. (2021). A tough act to follow: Collagen hydrogel modifications to improve mechanical and growth factor loading capabilities. Mater. Today Bio.

[125] Caliari S.R., Burdick J.A. (2016). A practical guide to hydrogels for cell culture. Nat. Methods.

[126] Luo X., Fong E.L.S., Zhu C., Lin Q.X.X., Xiong M., Li A., Li T., Benoukraf T., Yu H., Liu S. (2021). Hydrogel-based colorectal cancer organoid co-culture models. Acta Biomater..

[127] Samani A., Zubovits J., Plewes D. (2007). Elastic moduli of normal and pathological human breast tissues: An inversion-technique-based investigation of 169 samples. Phys. Med. Biol..

[128] Werner S., Grose R. (2003). Regulation of Wound Healing by Growth Factors and Cytokines. Physiol. Rev..

[129] Hospodiuk M., Dey M., Sosnoski D., Ozbolat I.T. (2017). The bioink: A comprehensive review on bioprintable materials. Biotechnol. Adv..

[130] Chen H., Cheng Y., Wang X., Wang J., Shi X., Li X., Tan W., Tan Z. (2020). 3D printed in vitro tumor tissue model of colorectal cancer. Theranostics.

[131] Alépée N., Bahinski A., Daneshian M., De Wever B., Fritsche E., Goldberg A., Hansmann J., Hartung T., Haycock J., Hogberg H. (2014). State-of-the-art of 3D cultures (organs-on-a-chip) in safety testing and pathophysiology. ALTEX.

[132] Pinho D., Santos D., Vila A., Carvalho S. (2021). Establishment of Colorectal Cancer Organoids in Microfluidic-Based System. Micromachines.

[133] Wishart A.L., Conner S.J., Guarin J.R., Fatherree J.P., Peng Y., McGinn R.A., Crews R., Naber S.P., Hunter M., Greenberg A.S. (2020). Decellularized extracellular matrix scaffolds identify full-length collagen VI as a driver of breast cancer cell invasion in obesity and metastasis. Sci. Adv..

[134] Lv Y., Wang H., Li G., Zhao B. (2021). Three-dimensional decellularized tumor extracellular matrices with different stiffness as bioengineered tumor scaffolds. Bioact. Mater..

[135] Liu G., Wang B., Li S., Jin Q., Dai Y. (2019). Human breast cancer decellularized scaffolds promote epithelial-to-mesenchymal transitions and stemness of breast cancer cells in vitro. J. Cell. Physiol..

[136] Leiva M.C., Garre E., Gustafsson A., Svanström A., Bogestål Y., Håkansson J., Ståhlberg A., Landberg G. (2021). Breast cancer patient-derived scaffolds as a tool to monitor chemotherapy responses in human tumor microenvironments. J. Cell. Physiol..

[137] Landberg G., Fitzpatrick P., Isakson P., Jonasson E., Karlsson J., Larsson E., Svanström A., Rafnsdottir S., Persson E., Gustafsson A. (2020). Patient-Derived Scaffolds Uncover Breast Cancer Promoting Properties of the Microenvironment. Biomaterials.

[138] Hoshiba T., Tanaka M. (2016). Decellularized matrices as in vitro models of extracellular matrix in tumor tissues at different malignant levels: Mechanism of 5-fluorouracil resistance in colorectal tumor cells. Biochim. Biophys. Acta (BBA)-Bioenerg..

[139] Hoshiba T., Tanaka M. (2015). Optimization of the tissue source, malignancy, and initial substrate of tumor cell-derived matrices to increase cancer cell chemoresistance against 5-fluorouracil. Biochem. Biophys. Res. Commun..

[140] Hoshiba T. (2018). An extracellular matrix (ECM) model at high malignant colorectal tumor increases chondroitin sulfate chains to promote epithelial-mesenchymal transition and chemoresistance acquisition. Exp. Cell Res..

[141] Chen H.J., Wei Z., Sun J., Bhattacharya A., Savage D.J., Serda R., Mackeyev Y., Curley S.A., Bu P., Wang L. (2016). A recellularized human colon model identifies cancer driver genes. Nat. Biotechnol..

[142] Nietzer S., Baur F., Sieber S., Hansmann J., Schwarz T., Stoffer C., Häfner H., Gasser M., Waaga-Gasser A.M., Walles H. (2016). Mimicking Metastases Including Tumor Stroma: A New Technique to Generate a Three-Dimensional Colorectal Cancer Model Based on a Biological Decellularized Intestinal Scaffold. Tissue Eng. Part C Methods.

[143] Alabi B.R., LaRanger R., Shay J.W. (2019). Decellularized mice colons as models to study the contribution of the extracellular matrix to cell behavior and colon cancer progression. Acta Biomater..

[144] Taylor D.A., Sampaio L.C., Ferdous Z., Gobin A.S., Taite L.J. (2018). Decellularized matrices in regenerative medicine. Acta Biomater..

[145] Park Y., Huh K.M., Kang S.-W. (2021). Applications of Biomaterials in 3D Cell Culture and Contributions of 3D Cell Culture to Drug Development and Basic Biomedical Research. Int. J. Mol. Sci..

[146] Dzobo K., Motaung K., Adesida A. (2019). Recent Trends in Decellularized Extracellular Matrix Bioinks for 3D Printing: An Updated Review. Int. J. Mol. Sci..

[147] Zhang W., Du A., Liu S., Lv M., Chen S. (2021). Research progress in decellularized extracellular matrix-derived hydrogels. Regen. Ther..

[148] Schanz J., Pusch J., Hansmann J., Walles H. (2010). Vascularised human tissue models: A new approach for the refinement of biomedical research. J. Biotechnol..

[149] Tian X., Werner M.E., Roche K.C., Hanson A.D., Foote H.P., Yu S.K., Warner S.B., Copp J.A., Lara H., Wauthier E.L. (2018). Organ-specific metastases obtained by culturing colorectal cancer cells on tissue-specific decellularized scaffolds. Nat. Biomed. Eng..

[150] Naba A., Clauser K.R., Ding H., Whittaker C.A., Carr S.A., Hynes R.O. (2016). The extracellular matrix: Tools and insights for the “omics” era. Matrix Biol..

[151] Parkinson G.T., Salerno S., Ranji P., Håkansson J., Bogestål Y., Wettergren Y., Ståhlberg A., Bexe Lindskog E.B., Landberg G. (2021). Patient-derived scaffolds as a model of colorectal cancer. Cancer Med..

[152] Aran D., Camarda R., Odegaard J., Paik H., Oskotsky B., Krings G., Goga A., Sirota M., Butte A.J. (2017). Comprehensive analysis of normal adjacent to tumor transcriptomes. Nat. Commun..

[153] Sanz-Pamplona R., Berenguer A., Cordero D., Molleví D.G., Crous-Bou M., Sole X., Paré-Brunet L., Guino E., Salazar R., Santos C. (2014). Aberrant gene expression in mucosa adjacent to tumor reveals a molecular crosstalk in colon cancer. Mol. Cancer.

[154] Ahmed E., Saleh T., Xu M. (2021). Recellularization of Native Tissue Derived Acellular Scaffolds with Mesenchymal Stem Cells. Cells.

[155] Gustafsson A., Garre E., Leiva M.C., Salerno S., Ståhlberg A., Landberg G. (2021). Patient-derived scaffolds as a drug-testing platform for endocrine therapies in breast cancer. Sci. Rep..

[156] Gharenaz N.M., Movahedin M., Mazaheri Z. (2021). Comparison of two methods for prolong storage of decellularized mouse whole testis for tissue engineering application: An experimental study. Int. J. Reprod. Biomed..

[157] Urbani L., Maghsoudlou P., Milan A., Menikou M., Hagen C.K., Totonelli G., Camilli C., Eaton S., Burns A., Olivo A. (2017). Long-term cryopreservation of decellularised oesophagi for tissue engineering clinical application. PLoS ONE.

